# Proteomic identification of the oncoprotein STAT3 as a target of a novel Skp1 inhibitor

**DOI:** 10.18632/oncotarget.13153

**Published:** 2016-11-07

**Authors:** Xin Cheng, Yong-Qiang Liu, Gui-Zhen Wang, Li-Na Yang, Yong-Zhi Lu, Xin-Chun Li, Bo Zhou, Li-Wei Qu, Xiao-Lu Wang, Yong-Xian Cheng, Jinsong Liu, Sheng-Ce Tao, Guang-Biao Zhou

**Affiliations:** ^1^ Division of Molecular Carcinogenesis and Targeted Therapy for Cancer, State Key Laboratory of Membrane Biology, Institute of Zoology, Chinese Academy of Sciences, Beijing 100101, China; ^2^ Shanghai Center for Systems Biomedicine, Key Laboratory of Systems Biomedicine (Ministry of Education), Shanghai Jiao Tong University, Shanghai 200240, China; ^3^ Guangzhou Institute of Biomedicine and Health, Chinese Academy of Sciences, Guangzhou 510530, China; ^4^ State Key Laboratory of Phytochemistry and Plant Resources in West China, Kunming Institute of Botany, Chinese Academy of Sciences, Kunming 650201, China

**Keywords:** proteome microarray, 6-O-angeloylplenolin, STAT3 inhibitor, Skp2, lung cancer

## Abstract

The S phase kinase-associated protein 1 (Skp1), an adaptor protein of the Skp1-Cul1-F-box protein complex, binds the ubiquitin E3 ligase Skp2 and is critical to its biological functions. Targeting of Skp1 by a small compound 6-O-angeloylplenolin (6-OAP) results in dissociation and degradation of Skp2 and mitotic arrest of lung cancer cells. Here, by using a proteome microarray containing 16,368 proteins and a biotinylated 6-OAP, we identified 99 proteins that could bind 6-OAP, with Skp1 and STAT3 sitting at the central position of the 6-OAP interactome. 6-OAP formed hydrogen bonds with Ser611/Ser613/Arg609 at the SH2 domain of STAT3 and inhibited the constitutive and interleukin-6-induced phosphorylated STAT3 (pSTAT3), leading to inhibitory effects on lung cancer cells and suppression of *Skp2* transcription. STAT3 was overexpressed in tumor samples compared to counterpart normal lung tissues and was inversely associated with prognosis of the patients. 6-OAP inhibited tumor growth in SCID mice intravenously injected with lung cancer cells, and downregulated both STAT3 and Skp2 in tumor samples. Given that 6-OAP is a Skp1 inhibitor, our data suggest that this compound may target Skp1 and STAT3 to suppress Skp2, augmenting its anti-lung cancer activity.

## INTRODUCTION

Lung cancer is the malignant epithelial tumor arising from the respiratory mucosa (bronchi, bronchioles, and alveoli) with complex changes across the genome [1–3]. It is the No. 1 cancer killer worldwide with an only 15% of five-year overall survival rate for all stages combined [4]. Targeted therapies such as epidermal growth factor receptor (EGFR) inhibitors benefit a proportion of patients [5, 6]), but will eventually fail because of drug resistance resulting from secondary mutations (e.g., T790M) in EGFR or other mechanisms [7]. The efficacy of immune checkpoint inhibitors is promising, and the long-term efficacy is under investigation [8, 9]. To further improve clinical outcome, combinatory treatment regimens or drugs perturbing multiple targets are desired [10, 11]. For example, arsenic trioxide which achieves a 5-year overall survival of more than 90% in acute promyelocytic leukemia when used in combination with retinoic acid, targets PML-RARα fusion protein, hexokinase-2, and binds 360 proteins [12, 13].

Cell cycle, the sequence of cellular transformations that accompany transition from one mitotic cell division to another, is a critical regulator of cell growth, proliferation and survival, and is stringently controlled by two types of E3 ligase complexes, the anaphase-promoting complex (APC) and the Skp1-Cullin-F-box protein (SCF) complex [14]. Termination of mitotic progression by microtubule inhibitors such as taxanes represents one of the most successful therapeutic approaches in cancer, but severe side effects and development of drug resistance limit their clinical use [15, 16]. To bypass these limitations, novel anti-mitotic strategies, e.g., suppression of Skp2 and Cdc20, have been investigated [17–19]. We recently showed that sequestration of Skp1 by occupying the sites critical to Skp1-Skp2 interaction by a natural compound 6-*O*-angeloylplenolin (6-OAP), caused dissociation and proteolysis of oncogenic E3 ligases Skp2, NIPA, and β-TRCP, and accumulation of their substrates Cyclin B1, P27 and E-Cadherin. 6-OAP induced mitotic arrest and exerted potent anti-lung cancer and anti-myeloma activity in murine models with low adverse effect and favorable pharmacological features [20, 21], suggesting the significant therapeutic potentials of this medicinal herb-derived natural compound.

In this study, we used a biotinylated 6-OAP (Bio-6-OAP) and a proteome microarray containing 16,368 proteins to systematically identify the direct targets of this compound. The results showed that 6-OAP bound 99 proteins with Skp1 and Signal transducer and activator of transcription 3 (STAT3) sitting at the central position of the signal network. 6-OAP inhibited constitutively activated- and IL-6-induced STAT3 activity, and suppressed STAT3-dependent *Skp2* transcription. Therefore, 6-OAP inhibited both Skp1 and STAT3 to repress Skp2, exhibiting inhibitory effects on lung cancer cell proliferation and survival.

## RESULTS

### Proteomic identification of 6-OAP binding proteins

**Figure 1:**
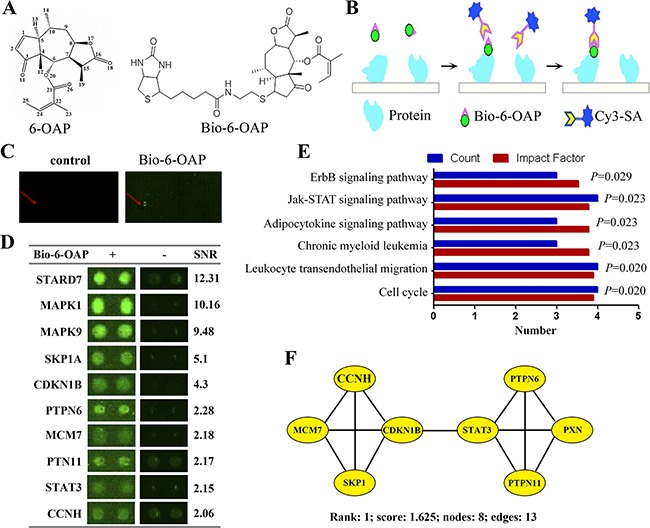
Identification of 6-OAP binding proteins. **A.** Chemical structure of 6-OAP and Bio-6-OAP. **B.** A schematic representation of identification of Bio-6-OAP binding protein using a proteome microarray and Bio-6-OAP. **C.** Images of two randomly picked blocks from the same location of both the biotin control (left) and the experimental microarrays (right). **D.** Images of 10 representatives of Bio-6-OAP binding proteins in the proteome microarray. **E.** KEGG analysis of pathways in 6-OAP binding proteins. **F.** The representative 6-OAP interactome. Proteins related to SKP1 and STAT3 are connected in a dense protein-protein interaction network that forms the densest cluster.

To uncover 6-OAP binding proteins, Bio-6-OAP (Figure 1A) was synthesized [20] and a human proteomic microarray containing 16,368 affinity purified N-terminal GST tagged proteins [22] was employed. Bio-6-OAP retained the anti-lung cancer activity and the mechanism of action of 6-OAP [20]. Bio-6-OAP or biotin was probed on the human proteome microarray, and after free Bio-6-OAP/biotin was removed, the microarray was further incubated with a Cy3 conjugated streptavidin (Cy3-SA) to present the Bio-6-OAP-protein interactions, and the specific binding between biotin and streptavidin was used for readout (Figure 1B). Two randomly picked blocks from the same location of both the experimental and control microarrays were compared and positive spots were identified (Figure 1C). The signal to noise ratio (SNR) for each spot was defined as the ratio of (median foreground minus median background) to standard deviation of median background, and the SNR of a protein was averaged from the two duplicated spots on each microarray. To call the candidates, the cutoff was set as SNR≥2, and after removal of nonspecific signal as compared to the vehicle control, 99 proteins were identified as potential target proteins of Bio-6-OAP (Supplementary Table S1). Representative spots of candidate proteins were shown in Figure 1D.

### Identification of SKP1 and STAT3 as key targets of 6-OAP

The 6-OAP targeting proteins was analyzed by using DAVID bioinformatics resources [23], and the results showed that the candidates were substantially enriched in cellular response to stress, JNK cascade, stress-activated protein kinase and intracellular signaling cascade (Supplementary Figure S1A). For molecular function, the most significant ones (*P*<0.01) were catalytic activity, protein binding and transferase activity (Supplementary Figure S1B). Pathway analysis was performed using the KEGG (Kyoto Encyclopedia of Genes and Genomes) analysis, and the results showed that molecules in cell cycle (SKP1, CDKN1B, MCM7, CCNH), leukocyte transendothelial migration (PXN, OCLN, PTPN11, PECAM1), chronic myeloid leukemia (PTPN11, CRKL, CDKN1B), adipocytokine signaling pathway (PTPN11, STAT3, MAPK9), and Jak-STAT signaling pathway (STAT3, PTPN11, PTPN6, CNTFR) were mostly significantly perturbed (Figure 1E). The 6-OAP interactome for highly-connected regions was analyzed by using a Cytoscape plugin MCODE [24], and prominent, highly-connected clusters formed by several complexes and cellular functions were revealed (Supplementary Figure S1C). Interestingly, proteins related to SKP1 and STAT3 are connected in a dense protein-protein interaction network that forms the densest cluster (Cluster 1) (Supplementary Figure S1C and Figure 1F), suggested that SKP1 and STAT3 may be the most important targets of 6-OAP.

### STAT3 is a direct target of 6-OAP

**Figure 2:**
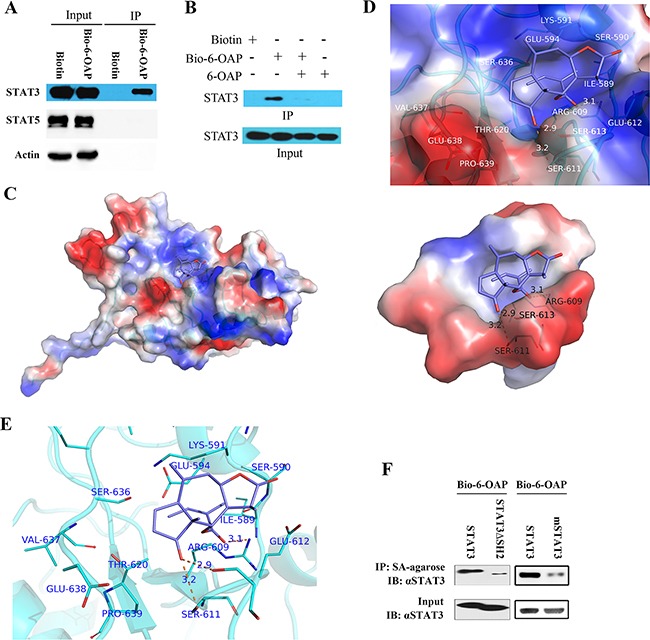
STAT3 is a direct target of 6-OAP **A.** H1975 cells were treated with Biotin or Bio-6-OAP at 50 μM for 6 hours, lysed, and the cell lysates were subjected to immunoprecipitation using streptavidin agarose and Western blot using indicated antibodies. **B.** H1975 cells were treated with Bio-6-OAP (50 μM) in the presence or absence of 6-OAP (100 μM) for 6 hours, lysed, and the cell lysates were subjected to immunoprecipitation using streptavidin agarose and Western blot. **C-E**. Computational modeling of 6-OAP docking to the SH2 domain of Stat3. (C) STAT3 and 6-OAP are shown as cartoon and sticks, respectively. Solvent-accessible surface of SH2 domain is shown. It is color coded according to the electrostatic potential, with red and blue indicating negative and positive, respectively. (D) Solvent-accessible surface of SH2 domain is shown color-coded according to the electrostatic potential, with red and blue indicating negative and positive, respectively. SH2 domain and 6-OAP are shown as cartoon and sticks, respectively. Hydrogen bonds binding to S611, S613 and R609 are shown as dash lines. (E) Interaction between 6-OAP and the SH2 domain of STAT3 (PDB code: 1BG1). SH2 domain was shown as cartoon; 6-OAP and residues at active site were shown as sticks. **F.** The 293T cells were transfected with SH2 domain-deleted STAT3 (STAT3ΔSH2) or STAT3 with S611A/S613A/R609A mutations (mSTAT3), treated with 50 μM Bio-6-OAP for 6 hours, lysed, the lysates were subjected to immunoprecipitation using streptavidin (SA) agarose and Western blot using indicated antibodies.

The above results confirmed our previous finding that SKP1 is a direct target of 6-OAP which binds Skp1 at sites critical to Skp1-Skp2 interaction, leading to dissociation and proteolysis of oncogenic E3 ligases NIPA, Skp2, and β-TRCP, and accumulation of their substrates Cyclin B1, P27 and E-Cadherin [20]. In this work, STAT3 was selected as a protein of interest for further investigation. To confirm 6-OAP/STAT3 interaction, the H1975 cells were treated with Bio-6-OAP for 6 hours, lysed, and proteins were subjected to Western blot assay. We found that STAT3, but not STAT5, was pulled down by streptavidin agarose (Figure 2A), suggesting the binding between 6-OAP and STAT3. Moreover, the binding of Bio-6-OAP with STAT3 could be attenuated markedly by the competition of unlabeled 6-OAP (Figure 2B).

Computational modeling of 6-OAP/STAT3 interaction showed that this compound binds STAT3 at its Src homology-2 (SH2) domain (Figure 2C) which is required for phosphorylation and dimerization of STAT3 [25]. SH2 domain had been shown to be targeted by a small compound S31-201 which forms hydrogen bonding with Lys-591, Ser-611, Ser-613, and Arg-609 [26]. We found that 6-OAP binds SH2 by forming hydrogen bonds with Ser611, Ser613 and Arg609 (Figure 2D, E). To confirm the significance of SH2 and S611/S613/R609 in binding with 6-OAP, expression plasmids of *STAT3* with SH2 domain depletion (STAT3ΔSH2) or mutations in Ser611/Ser613/Arg609 (S611A/S613A/R609A; designated mSTAT3) were constructed and transfected into 293 cells which were treated with Bio-6-OAP for additional 6 hours. The cells were then lysed and the lysates were incubated with Streptavidin (SA)-agarose and detected by Western blot. We found that while the wild type STAT3 showed high binding affinity with Bio-6-OAP, deletion of SH2 or mutations in S611/S613/R609 markedly attenuated the binding affinity (Figure 2F).

### 6-OAP inhibits constitutive and interleukin-6 (IL-6)-induced STAT3 activity

**Figure 3:**
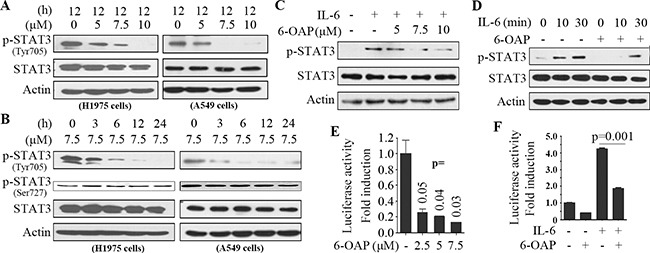
6-OAP is a STAT3 inhibitor **A.** The cells were treated with 6-OAP at indicated concentrations for 12 hours, lysed, and Western blot was performed using indicated antibodies. **B.** The cells were treated with 6-OAP at 7.5 μM for indicated time points, lysed, and Western blot was performed. **C.** A549 cells were serum starved for 6 hours, treated with IL-6 at 10 ng/ml for 1 hour, followed by treatment with 6-OAP for 3 hours, lysed, and Western blot was performed. **D.** A549 cells were pretreated with 6-OAP at 7.5 μM for 3 hours, followed by stimulation with IL-6 at 10 ng/ml for indicated time points. Whole-cell extracts were examined by Western blot. **E.** Luciferase assays were performed using A549 cells transfected with pAPRE-luc and pRL-CMV (as an internal control), and treated with 6-OAP for 24 hours. **F.** A549 cells were transfected with pAPRE-luciferase and pRL-CMV, serum starved for 6 hours, and treated with 6-OAP (7.5 μM) or IL-6 (30 ng/ml) for 24 hours, and relative luciferase activities were normalized with the internal control.

STAT3 is a transcription factor that regulates genes involved in cell growth, proliferation, and survival, and is activated by phosphorylation by upstream Janus activated kinases (JAKs) and the interleukin (IL)-6 family cytokines and is inactivated by dephosphorylation [27, 28]. We tested the effect of 6-OAP on STAT3 phosphorylation, and found that this compound inhibited phosphorylation of STAT3 (at Tyr705 but not Ser727) in a dose- and time-dependent manner in H1975 and A549 cells (Figure 3A, B). In A549 cells, treatment with IL-6 at 10 ng/ml for 1 hour up-regulated pSTAT3, while incubation with 6-OAP at 5 to 10 μM for 3 hours markedly antagonized this effect (Figure 3C). In line with these observations, pretreatment with 6-OAP at 7.5 μM for 3 hours (and then washed out the drug) drastically slowed down or inhibited IL-6 (10 ng/ml)-induced phosphorylation of STAT3 (Figure 3D).

To test the effect of 6-OAP on STAT3 transcriptional activity, A549 cells were transfected with the reporter plasmid pAPRE-luciferase (containing STAT3-specific binding element APRE [29]) and treated with 6-OAP for 24 hours, and luciferase reporter assays were performed. We found that 6-OAP at 7.5 μM suppressed the luciferase activity of up to 85% (Figure 3E). Moreover, while IL-6 (10 ng/ml) significantly increased STAT3 transcriptional activity, 6-OAP antagonized this effect (Figure 3F).

### Transcriptional suppression of STAT3 target gene Skp2

**Figure 4:**
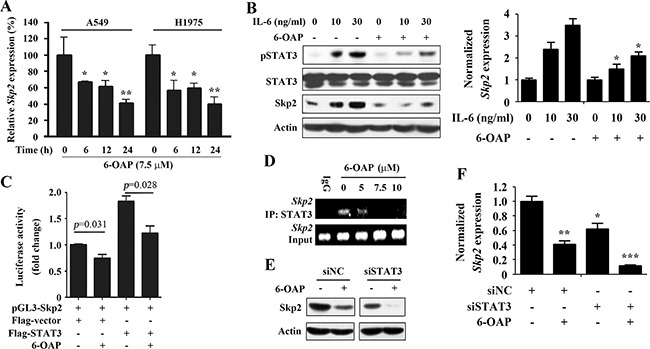
Inhibition of STAT3 mediates 6-OAP-induced suppression of Skp2 transcription **A.** A549 and H1975 cells were treated with 6-OAP at 7.5 μM for indicated time points, lysed, and the expression of *Skp2* was detected by real-time RT-PCR. **B.** A549 cells were serum starved, pretreated with 6-OAP at 7.5 μM for 3 hours (then washed out the drug), followed by stimulation with IL-6 at 10 – 30 ng/ml for 1 hour. The cells were lysed, and Skp2 expression was examined by Western blot (left) and real-time RT-PCR (right). *, P<0.05. **C.** Luciferase assays using 293T cells expressing Flag-STAT3 and/or pGL3-Skp2 reporter and pRL-TK (as an internal control), with or without 6-OAP treatment (7.5 μM for 24 hours). **D.** A549 cells were treated with 6-OAP for 24 hours, lysed, and chromatin immunoprecipitation (ChIP) assay was performed using an anti-STAT3 antibody and *Skp2* expression was detected by RT-PCR. **E, F**. A549 cells were transfected with siNC or si*STAT3* for 24 hours, and treated with or without 6-OAP (at 7.5 μM for 24 hours). The cells were lysed and the expression of Skp2 was tested by Western blot (E) and real-time RT-PCR (F). *, P<0.05; **, P<0.01; ***, P<0.001.

A previous study demonstrated that Skp2 is a target of STAT3 [30], and our previous work showed that Skp2 is dissociated from Skp1 and underwent proteolysis in lung cancer cells treated with 6-OAP [20]. We tested the effect of 6-OAP on *Skp2* expression at mRNA level, and reported that in A549 and H1975 cells 6-OAP at 7.5 μM significantly inhibited *Skp2* expression in a time-dependent manner (Figure 4A). In A549 cells, IL-6 (10 ng/ml) up-regulated pSTAT3 whereas 6-OAP suppressed this up-regulation at both protein and mRNA levels (Figure 4B). We found that IL-6 also induced upregulation of Skp2, which was suppressed by 6-OAP (Figure 4B). In 293T cells transfected with pGL3-Skp2 reporter plasmid, 6-OAP inhibited Skp2 luciferase activity; exogenous STAT3 significantly increased Skp2 luciferase activity, whereas 6-OAP antagonized this phenomenon (Figure 4C). In a chromatin immunoprecipitation (ChIP) assay using an anti-STAT3 antibody, we showed that *Skp2* was enriched by STAT3 in A549 cells, whereas 6-OAP repressed STAT3-*Skp2* binding affinity (Figure 4D). We further showed that in cells transfected with siNC, treatment with 6-OAP at 7.5 μM for 24 hours induced downregulation of Skp2, whereas silencing of STAT3 by siRNA enhanced 6-OAP-caused downregulation of Skp2 at both protein (Figure 4E) and mRNA (Figure 4F) levels.

### The expression of STAT3 and Skp2 in lung cancer

**Figure 5:**
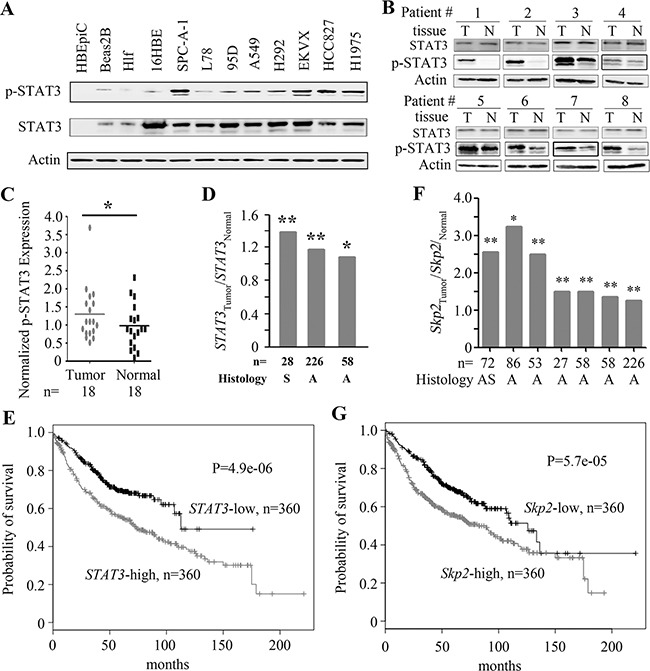
The expression of STAT3 and Skp2 in lung cancer **A.** STAT3 in normal and cancerous cells was detected by Western blot. **B, C**. The expression of pSTAT3 in tumor and counterpart normal lung tissues isolated from NSCLCs was tested by Western blot **(B)** and the results were further evaluated by densitometry analysis **(C)**. **D.** *STAT3* expression was detected by microarrays in tumor samples and normal lung tissues. The data were obtained from the Oncomine database. S, Squamous cell carcinoma; A, Adenocarcinoma. **E.** Overall survival of the adenocarcinoma patients with high or low level *STAT3*. The data were obtained from a database at http://kmplot.com/analysis/index.php?p=service&cancer=lung, and the expression of *STAT3* was detected by microarray. **F.** *Skp2* expression in NSCLCs, detected by microarray. The data were obtained from the Oncomine database. **G.** Overall survival of NSCLC patients with high or low level *Skp2*. The data were obtained from a database at http://kmplot.com/analysis/index.php?p=service&cancer=lung, and the expression of *Skp2* was detected by microarray. *, P<0.05; **, P<0.01; ***, P<0.001.

We tested the expression of STAT3 in several cell lines by Western blot, and found that the expression of pSTAT3 in lung cancer lines (SPC-A-1, EKVX, HCC827, H1975, H292, A549, 95D, and L78) was higher than in normal bronchial epithelial (HBEpiC, Beas-2B, 16HBE) or lung fibroblast (HLF) cell lines (Figure 5A). In 18 previously untreated NSCLCs the expression of pSTAT3 was also significantly higher in tumor samples than their adjacent normal lung tissues in 8 (44.4%) patients (Figure 5B, C). In 3 studies documented in Oncomine data base [31–33], the expression of *STAT3* at mRNA level (detected by microarray) in tumor samples was significantly higher than in counterpart normal lung tissues (Figure 5D). We further tested the potential relationship between the *STAT3* expression and life span of the patients using data deposited in a website (http://kmplot.com/analysis/index.php?p=service&start=1) [34], and found that the expression of *STAT3* was inversely associated with survival of patients with lung adenocarcinoma (Figure 5E). In works of Oncomine data base [32, 33, 35–39], the expression of *Skp2* at mRNA level in tumor samples was significantly higher than in counterpart normal lung tissues (Figure 5F). In gene expression profiles and survival data base deposited in a website (http://kmplot.com/analysis/index.php?p=service&start=1) [34], the expression of *Skp2* was inversely associated with overall survival of NSCLC patients (Figure 5G).

### 6-OAP inhibits STAT3 and suppresses lung cancer in vivo

**Figure 6:**
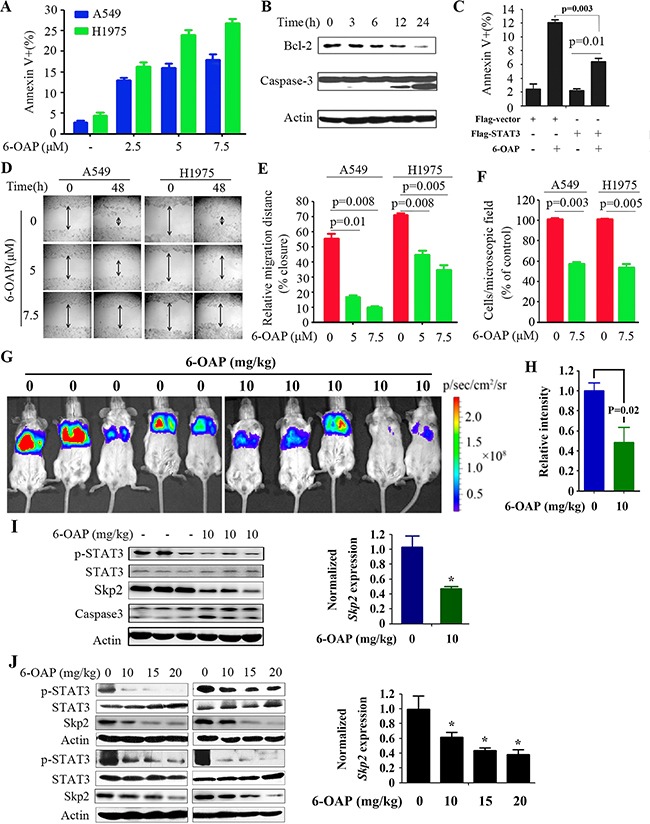
Inhibitory effects of 6-OAP on lung cancer cells in vitro and in vivo **A.** A549 and H1975 cells were treated with 6-OAP at indicated concentrations for 24 hours. The cells were analyzed by Annexin V/propidium iodide (PI) staining and flow cytometry. **B.** H1975 cells were treated with 6-OAP at 7.5 μM for indicated time points, lysed, and Western blot was performed. **C.** H1975 cells were transfected with flag-STAT3 or vector control, and 24 hours later treated with 6-OAP. The cells were analyzed by Annexin V/PI staining and flow cytometry. **D, E**. Wound healing assays of A549 and H1975 cells upon 6-OAP treatment. Cultures were imaged and visualized at 10× magnification by light microscopy **(D)**. Data represent the means ± SD from three independent experiments (**E)**. **F.** Results of transwell assays. The cells were seeded into transwell chambers and treated with 6-OAP. After 24 hours, migrated cells stained with 0.1% crystal violet were counted. Data represent the means ± SD from three independent experiments. **G.** A549-Luciferase cells were intravenously injected into SCID mice, and 1 week later the mice were randomized to receive vehicle (n = 6) or 6-OAP treatment (n = 8). The mice were detected by IVIS Spectrum. **H.** The relative luciferase intensity in the mice. **I.** Western blot (left) and real-time RT-PCR (right) analyses were performed using lysates of tumor samples isolated from the SCID mice. **J.** Western blot (left) and real-time RT-PCR (right) analyses were performed using lysates of tumor samples isolated from the nude mice subcutaneously inoculated with H1975 cells. *, P<0.05.

6-OAP is a cell cycle inhibitor that arrests mitosis [20, 21]. Here we showed 6-OAP also moderately induced apoptosis in lung cancer cells. Treatment with 6-OAP at 7.5 μM for 24 hours resulted in apoptosis in 18% of A549 and 27% of H1975 cells (Figure 6A). In these cells, the apoptosis suppressors Bcl-2 was downregulated whereas Caspase-3 was activated by 6-OAP treatment (Figure 6B). In A549 cells treated with 6-OAP, the exogenous STAT3 partially rescued 6-OAP-induced programmed cell death (Figure 6C), suggesting a role of STAT3 inhibition in 6-OAP-induced apoptosis. We further showed that in a wound-healing experiment, 6-OAP inhibited lung cancer cells to migrate into the wound gap (Figure 6D, E), and suppressed cell migration in a transwell chamber migration assay (Figure 6F).

We showed that in SCID mice injected with A549-luciferase cells (1×10^6^) via tail vein, treatment with 10 mg/kg 6-OAP (once every two days for 30 days) significantly suppressed tumor growth (Figure 6G, H), in consistent with our previous report that 6-OAP (20 mg/kg) significantly suppressed tumor growth in SCID mice bearing A549-luciferase cells and in nude mice subcutaneously inoculated with H1975 cells [20]. We tested the effect on 6-OAP on STAT3 *in vivo* by Western blot analysis of lysates of tumor samples isolated from the mice, and found that treatment with 10 mg/kg 6-OAP drastically inhibited pSTAT3 in tumor samples (Figure 6I). 6-OAP at 10 – 20 mg/kg also repressed pSTAT3 in tumor samples of nude mice inoculated with H1975 cells (Figure 6J). Moreover, 6-OAP downregulated Skp2 at both mRNA and protein levels in these samples (Figure 6I, J).

## DISCUSSION

STAT3, a member of the STATs family that regulates gene transcription through relaying signals from activated plasma membrane receptors to the nucleus, is activated by the phosphorylation of Tyr705 by upstream kinases such as JAK2 and the subsequent formation of dimerization of STAT3 monomers via their SH2 domains [40, 41]. STAT3 acts as an oncogene [42] and promotes proliferation, survival [43], angiogenesis, and metastasis [44], and interferes with apoptosis and anti-tumor immune responses [28]. Constitutive activation of STAT3 has been demonstrated in most human cancers including breast cancer [45], leukemia and lymphoma [46], multiple myeloma [47], and gastric cancer [48], and is associated with a poor prognosis in colorectal cancer [49], gastric cancer [50], and ovarian cancer [51]. STAT3 is persistently activated in about 50% of NSCLCs, especially in adenocarcinomas harboring activating mutations in EGFR which activate the gp130/JAK/STAT3 pathway by means of IL-6 upregulation [52, 53]. We showed that in 8/18 (44.4%) NSCLCs, pSTAT3 was higher in tumor samples than in counterpart normal lung tissues (Figure 5B, C). Moreover, *STAT3* high expression was inversely associated with poor prognosis of the patients (Figure 5D). These results suggest that STAT3 may have an important role in lung carcinogenesis.

STAT3 is an attractive therapeutic target for both the early stages and metastatic disease. STAT3 inhibition strategies include inhibition of the STAT3 DNA binding domain, abrogation of the STAT3 N-terminal domain, suppression of the STAT3 SH2 domain, inhibition of the STAT3-importin interaction, and blockage of upstream kinase activity [28]. We showed that 6-OAP could bind the SH2 domain by forming hydrogen bonds with Ser611/Ser613/Arg609 (Figure 3C–3E), whereas depletion of SH2 domain or mutations in Ser611/Ser613/Arg609 abrogated STAT3-6-OAP interaction (Figure 3F). 6-OAP inhibited constitutively activated STAT3, suppressed IL-6-induced pSTAT3 (Figure 3), and triggered apoptosis and suppressed migration of lung cancer cells *in vitro* (Figure 6) and showed potent anti-lung cancer activity *in vivo* (Figure 6) [20]. In a rescue assay, exogenous expression of STAT3 partially suppressed 6-OAP-induced apoptosis of the cells (Figure 6C), suggesting that inhibition of STAT3 at least partially mediated the anti-lung cancer effect of 6-OAP.

High-throughput proteomic approach provides a powerful tool to systematically identify the targets of anti-cancer drugs. Using the proteomic microarray containing 16,368 proteins, 360 proteins that specifically bind arsenic were unveiled [13], indicating the significant capability of this method. Here by using this proteomic microarray and Bio-6-OAP, 99 candidate targets of 6-OAP were identified. Skp1 which has been shown to be a direct target of 6-OAP [20], was found to be one of the two key targets of this compound (Figure 1F), demonstrating the reproducibility and specificity of this strategy. Therefore, the proteomic approaches in combination with labeling technology will be helpful to uncover the direct targets of most compounds, including those once thought to have single or limited targets.

There is growing interest in developing therapies targeting SCF complexes which are multi-protein E3 ubiquitin ligase complexes catalyzing the ubiquitination and degradation of a variety of proteins. Skp2 is frequently overexpressed in many human cancers and plays a key role in tumorigenesis, whereas inhibition of Skp2 functions (either proteolytic function or non-proteolytic function) is emerging as a promising and novel anti-cancer strategy [54]. Recent studies showed that Skp1 was overexpressed in NSCLCs and was associated with poor prognosis [20]. Abnormal Skp1 was also found in breast cancers [55]. Targeting Skp1 by engineered ubiquitin variants at the Skp1–F-box interface for Cul1 binding site inhibit SCF ubiquitin ligases [56]. We showed that 6-OAP sequestrates Skp1 and dissociates Skp2, leading to proteolysis of the oncoprotein [20, 57]. Inactivation of STAT3 by 6-OAP also resulted in transcription suppression of *Skp2* (Figure 4), enhancing the inhibitory effect of 6-OAP on Skp2 to suppress lung cancer. These results indicate that a small compound can target multiple molecules to modulate signal cascade, contributing to its anti-cancer activity.

6-OAP is a sesquiterpene lactone extracted from a medicinal herb *Centipeda* minima (L.) which is rich in some East and South East Asia countries such as China, Korea, and Nepal [58]. *Centipeda minima* is a *Compositae* plant used by Chinese and Korea people as folk medicine to treat headache, cough, expectoration, nasal allergy, diarrhea, malaria, and asthma [20, 21]. 6-OAP exhibits inhibitory effects on human lung, colorectal, liver, stomach, skin cancer, and multiple myeloma cells, and shows low adverse effect and favorable pharmacological features [20, 21, 59]. Therefore, the anti-lung cancer efficacy of 6-OAP or its parental plant *Centipeda minima*, should be tested in clinical investigations.

## MATERIALS AND METHODS

### Chemicals and reagents

6-OAP was isolated from *Centipeda minima* by our chemistry group, and the purity of this compound reached 99.5% [59]. Labeling of 6-OAP was performed by Boshixing Synthetic Technologies, Inc. (Shenzhen, Guangdong, China) as described [20]. 6-OAP was dissolved in DMSO (Sigma-Aldrich, St. Louis, MO, USA) at a stock solution of 10^−2^ M and stored at -20°C. IL-6 was purchased from R&D Systems, Inc. (Minneapolis, MN, USA). The 3-(4, 5-Dimethylthiazol-2-yl)-2, 5-diphenyltetrazolium bromide (MTT) was purchased from Amresco Inc. (Solon, OH, USA). Biotin was obtained from Sigma-Aldrich.

### Antibodies

The antibodies used in this study were as follows: anti-pSTAT3 (Tyr705), anti-pSTAT3 (Ser727), anti-STAT3, anti-STAT5, anti-Caspase-3, goat anti-rabbit IgG-HRP and goat anti-mouse IgG HRP antibody (Cell Signaling Technology, Beverly, MA, USA); anti-Skp2, anti-Bcl-2 (Santa Cruz Biotechnology, Santa Cruz, CA, USA); anti-Flag M2, and anti-β-Actin (Sigma) antibodies.

### Cell culture

The lung cancer cell lines NCI-H1975, NCI-292, HCC827, A549 and human embryonic kidney HEK-293 cells were obtained from the American Tissue Culture Collection (ATCC, Manassas, VA, USA) and Human normal bronchial epithelial cell line 16HBE was purchased from the Cell Resource Center, Chinese Academy of Medical Sciences (Beijing). Normal human bronchial epithelial cells (HBEpiC, Catalog Number: 3210) were purchased from ScienCell (ScienCell Research Laboratories, San Diego, CA, USA). Human lung squamous carcinoma cell lines L78 and highly metastatic large-cell lung cancer cell lines 95D were obtained from the Cell bank of Chinese Academy of Sciences (Shanghai), and human embryonic lung fibroblast HLF cells were purchased from Kenqiang Instrument Co., Ltd (Shanghai). BEAS-2B bronchial epithelial cells were provided by Professor Hongbin Ji at Shanghai Institute for Biological Sciences, Chinese Academy of Sciences. A549, HLF and BEAS-2B cells were cultured in Dulbecco modified Eagle medium (DMEM) containing 10% fetal bovine serum (FBS; Gibco/BRL, Grand Island, NY), 100 U/ml penicillin, 100 mg/ml streptomycin. HBEpiC cells were cultured in a serum-free Bronchial Epithelial Cell Medium (BECM, Cat. No. 3211, ScienCell Research Laboratories) containing essential and non-essential amino acids, vitamins, hormones, growth factors and trace minerals. L78, 95D, HCC827, NCI-292, NCI-H1975 and 16HBE cells were cultured in RPMI 1640 supplemented with 10% fetal bovine serum (FBS; Gibco/BRL, Grand Island, NY), 100 U/ml penicillin, 100 mg/ml streptomycin.

### Assessment of cell proliferation, apoptosis and cell cycle

Cancer cells (5×10^3^) were cultured in each well of 96-well tissue culture plates (Coaster, Charlotte, NC) and incubated without or with 6-OAP/Bio-6-OAP for indicated concentrations at 37°C in a 5% CO2 atmosphere. MTT assay was performed as described [60]. The data were calculated using the Graphpad Prism software (version 5.01, Graphpad software, Inc., California, USA). Cell apoptosis was measured using Annexin V/PI Apoptosis Detection kit (BD Biosciences, San Jose, CA) according to the manufacturer's instructions. Cell cycle distribution was analyzed by flow cytometry (BD FACS Vantage Diva, USA) and CellQuest software (BD Biosciences, San Jose, CA).

### Cell migration assays and wound healing assay

Transwells with 8-μM pores were incubated at 37°C in a CO2 incubator for at least 1 hour. A549 and H1975 were added to the upper chamber and allowed to migrate for 24 hour. Cells on the inserts were fixed with 90% ethanol, stained with 0.0005% Gentian Violet Solution, and washed with PBS. Non-migrated cells on the upper side of the inserts were wiped off with a cotton swab. Migrated cells were counted in five microscopic fields at 4× magnification, and the counts were averaged.

### Proteome microarray assay and data analysis

Human proteome microarray fabrication was carried out as previously described [13]. Proteome microarrays were blocked with blocking buffer; Bio-6-OAP or biotin was diluted to 50 μM in blocking buffer and incubated on the blocked proteome microarray. The washed microarrays were incubated with Cy3-Streptavidin at 1:1000 dilution (Sigma, St Louis, MO), washes and spun dry and subjected for scanning with a Genepix 4200A (Axon Instruments, Sunnyvale, CA) to visualize and record the results. Data were extracted by GenePix Pro 6.0 from the microarray images. Protein interaction networks of the candidates proteins were built automatically by the STRING (Search Tool for the Retrieval of Interacting Genes/Proteins) system (http://string-db.org/). A network of protein-protein interactions was generated, visualized by Cytoscape v2.8.1 (http://www.cytoscape.org), and further analyzed for densely connected regions using a graph theoretic clustering algorithm “Molecular Complex Detection” (MCODE) [24].

### Molecular docking analysis

Molecule docking was carried out by the program Autodock Vina [61]. The SH2 domain of STAT3 from the complex crystal structure (PDB code: 1BG1) was used as receptor. Both receptor and ligand 6-OAP were prepared by AutoDock Tools [62]. The Autodock Vina exhaustiveness parameter and num_modes were set as 50 and 100, respectively.

### Immunoprecipitation and streptavidin agarose affinity assay

Cell pellets were lysed in NETN lysis buffer containing 50 mM Tris HCL (pH 7.4), 137 mM NaCl, 1% NP40, 2mM EDTA and protease inhibitors cocktail (Sigma). For immunoprecipitations, cells were lysed on ice for 30 minutes in NETN buffer. Lysates were centrifuged, the supernatant was incubated with indicated antibodies overnight at 4°C, after which protein A/G Plus beads (Santa Cruz Biotechnology) were added and incubated at 4°C for 4 h. The beads were washed 4 times in NETN buffer. Then the beads were resuspended in SDS-PAGE loading buffer and boiled for 5 min. Equal amounts of protein samples were separated by SDS-PAGE, transferred to nitrocellulose and immunoblotted with antibodies indicated. For Streptavidin agarose affinity assay, cells upon Bio-6-OAP were lysed, the cell lysates in NETN buffer were incubated with streptavidin agarose overnight at 4°C, then washed with lysis buffer and boiled in SDS-PAGE loading buffer. For 6-OAP competition, the cell lysates were pretreated with 6-OAP (100 μM) for 1 hour, followed by 50 μM Bio-6-OAP treatment for 3 hours at 4°C, and streptavidin agarose affinity assay were performed. Western blot assays were performed as described with indicated antibodies.

### Luciferase reporter assays

A549 cells were cotransfected with the reporter plasmid pAPRE-luc (containing the STAT3 responsive element, a gift kindly provided by Professor Zhijie Chang (University of Tsinghua, Beijing, China) and the internal control plasmid pRL-CMV with lipofectamine 2000 (Invitrogen, Frederick, MD, USA) according to the manufacturer's protocol. The cells were treated with IL-6 and 6-OAP for 24 hours, then firefly and Renilla luciferase activities were measured using the Dual-Luciferase Reporter Assay system (Promega, Fitchburg, WI, USA), and the relative reporter activity was normalized to the Renilla luciferase activity. Each assay was repeated in three independent experiments.

### Murine models

The animal studies were approved by the Institutional Review Board of Institute of Zoology, Chinese Academy of Sciences. All animal studies were conducted according to protocols approved by the Animal Ethics Committee of our institute. SCID mice (5–6 weeks old) were purchased from Vital River Laboratory Animal Technology (Beijing, China). The mice were injected with A549-luciferase cells (1×106) via tail vein, and randomized into 2 groups a week later to receive vehicle or 6-OAP treatment (10 mg/kg, once every two days for 30 days). The mice were imaged by the IVIS Spectrum In Vivo Imaging System (PerkinElmer, Waltham, MA, USA) at day 40, and were euthanized by cervical dislocation when they became moribund.

### Statistical analysis

All experiments were repeated at least three times and the data are presented as the mean ± SD unless noted otherwise. Differences between data groups were evaluated for significance using student t-test of unpaired data or one-way analysis of variance and Bonferroni post-test. The overall survival data of the patients were obtained from a database at http://kmplot.com/analysis/index.php?p=service&cancer=lung, and the survival curves were plotted according to Kaplan-Meier method and compared by log-rank test. P values <0.05 were considered statistically significant.

## SUPPLEMENTARY MATERIALS FIGURE AND TABLE




