# Supplementary material for: Proteomic identification of the oncoprotein STAT3 as a target of a novel Skp1 inhibitor
Source: Oncotarget. 2016 Nov 7;8(2):2681–93. doi: 10.18632/oncotarget.13153 (PMC5356833; doi:10.18632/oncotarget.13153)
Supplement: Supplementary file 1 [file oncotarget-08-2681-s001.pdf]

Proteomic identification of the oncoprotein STAT3 as a target of a novel Skp1 inhibitor

SUPPLEMENTARY FIGURE AND TABLE

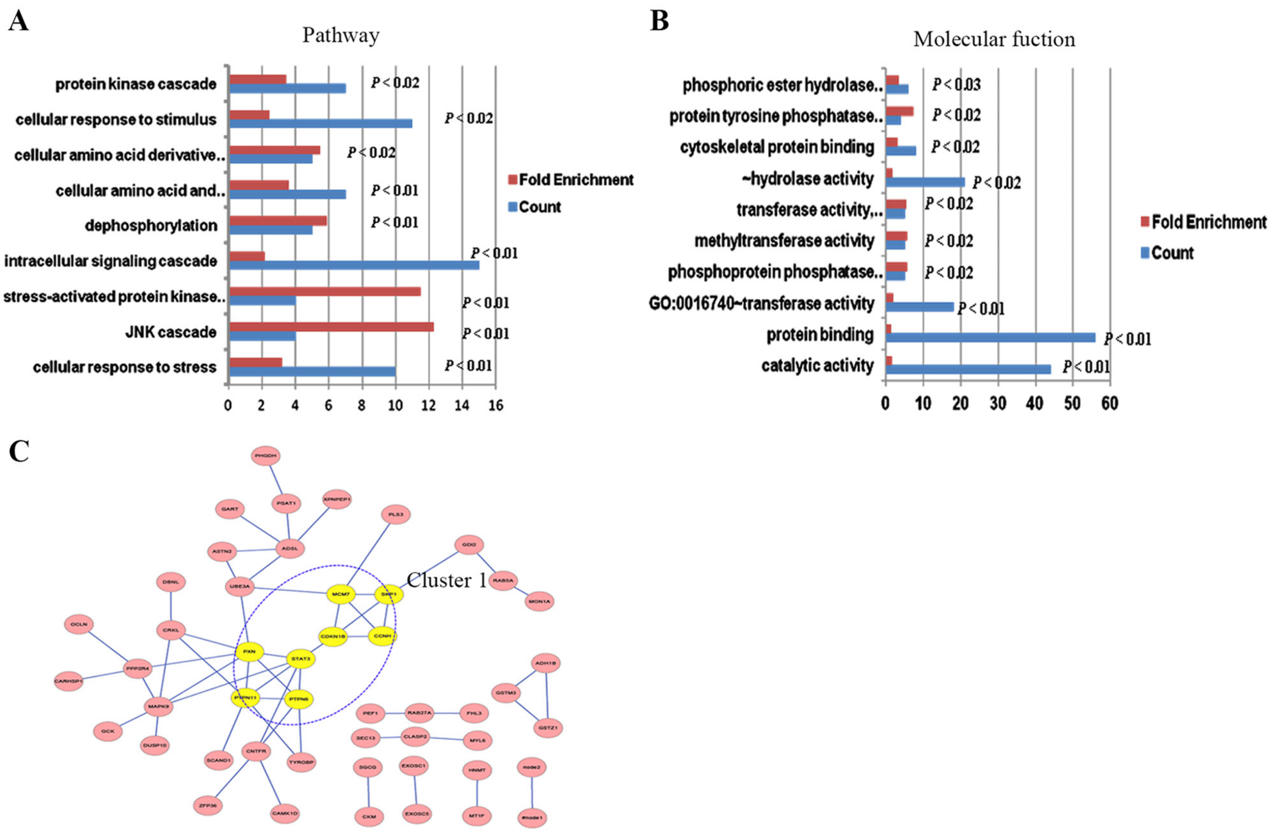

Supplementary Figure S1: Analysis of 6-OAP binding proteins. A. Biological process GO analysis of direct targets of 6-OAP, the top 9 GO term were shown. **B.** Molecular function GO analysis of direct targets of 6-OAP, the top 10 GO terms were shown. **C.** Interactome of 6-OAP binding proteins shows highly-connected regions using a Cytoscape plugin MCODE (Rank: 1, Score: 1.625, Nodes: 8, Edges: 13).

Supplementary Table S1: The 99 6-OAP-binding proteins.

See Supplementary File 1
