# Supplementary material for: Proteomic identification of the oncoprotein STAT3 as a target of a novel Skp1 inhibitor
Source: Oncotarget. 2016 Nov 7;8(2):2681–93. doi: 10.18632/oncotarget.13153 (PMC5356833; doi:10.18632/oncotarget.13153)
Supplement: Supplementary file 2 [file oncotarget-08-2681-s002.doc]

**Table S1. The 99 6-OAP-binding proteins**.

| No. | Symbol | SNR |
| --- | --- | --- |
| 1 | MT1F | 23.02 |
| 2 | STARD7 | 12.31 |
| 3 | MAPK1IP1L | 10.16 |
| 4 | MAPK9 | 9.48 |
| 5 | CAMK1D | 8.34 |
| 6 | CARHSP1 | 7.69 |
| 7 | FNDC3B | 6.90 |
| 8 | SLC6A18 | 6.71 |
| 9 | NUBP2 | 6.48 |
| 10 | RAB27A | 6.22 |
| 11 | SCAND1 | 6.06 |
| 12 | CLASP2 | 5.90 |
| 13 | CCDC97 | 5.18 |
| 14 | SKP1A | 5.1 |
| 15 | ATG4B | 4.94 |
| 16 | PEF1 | 4.77 |
| 17 | PDCL | 4.73 |
| 18 | RABEPK | 4.73 |
| 19 | FHL3 | 4.71 |
| 20 | ARF4 | 4.71 |
| 21 | PGM3 | 4.67 |
| 22 | HCG3 | 4.60 |
| 23 | ADSL | 4.49 |
| 24 | ECE2 | 4.39 |
| 25 | CDKN1B | 4.30 |
| 26 | ARIH2 | 4.10 |
| 27 | MPST | 4.05 |
| 28 | XPNPEP1 | 3.98 |
| 29 | ASTN2 | 3.88 |
| 30 | PLS3 | 3.80 |
| 31 | ID3 | 3.74 |
| 32 | GLTPD1 | 3.55 |
| 33 | CKM | 3.55 |
| 34 | TYROBP | 3.37 |
| 35 | DNAJA4 | 3.37 |
| 36 | HNMT | 3.33 |
| 37 | PHGDH | 3.29 |
| 38 | IGLC1 | 3.24 |
| 39 | GCK | 3.22 |
| 40 | SGCG | 3.20 |
| 41 | RAB5A | 3.20 |
| 42 | GART | 3.19 |
| 43 | EXOSC5 | 3.19 |
| 44 | HENMT1 | 3.16 |
| 45 | DBNDD2 | 3.14 |
| 46 | NUDT12 | 3.08 |
| 47 | SPRR1B | 2.93 |
| 48 | DBNL | 2.92 |
| 49 | TPMT | 2.90 |
| 50 | CRKL | 2.87 |
| 51 | PTPRE | 2.82 |
| 52 | ADH1B | 2.81 |
| 53 | ZNF223 | 2.81 |
| 54 | C19orf57 | 2.8 |
| 55 | CNTFR | 2.77 |
| 56 | PHPT1 | 2.77 |
| 57 | ACTR3B | 2.76 |
| 58 | MYL6 | 2.73 |
| 59 | C18orf56 | 2.71 |
| 60 | NUDCD2 | 2.70 |
| 61 | UBE3A | 2.7 |
| 62 | C12orf10 | 2.67 |
| 63 | TES | 2.65 |
| 64 | GSTZ1 | 2.63 |
| 65 | THRAP6 | 2.61 |
| 66 | OCLN | 2.61 |
| 67 | PREP | 2.57 |
| 68 | MON1A | 2.56 |
| 69 | CPNE1 | 2.5425 |
| 70 | ODC1 | 2.53 |
| 71 | EXOSC1 | 2.51 |
| 72 | PECAM1 | 2.47 |
| 73 | GDI2 | 2.42 |
| 74 | PXN | 2.42 |
| 75 | TRPV4 | 2.38 |
| 76 | RPS21 | 2.3 |
| 77 | PTPN6 | 2.28 |
| 78 | DHX40 | 2.26 |
| 79 | OVOL2 | 2.25 |
| 80 | STK16 | 2.24 |
| 81 | C2orf27B | 2.24 |
| 82 | ZFP36 | 2.22 |
| 83 | MCM7 | 2.18 |
| 84 | PTPN11 | 2.17 |
| 85 | SQSTM1 | 2.17 |
| 86 | GPKOW | 2.16 |
| 87 | TMEM103 | 2.15 |
| 88 | STAT3 | 2.15 |
| 89 | AKR1C4 | 2.15 |
| 90 | DUSP10 | 2.14 |
| 91 | SEC13 | 2.10 |
| 92 | GSTM3 | 2.07 |
| 93 | CCNH | 2.06 |
| 94 | KLHL2 | 2.06 |
| 95 | TTRAP | 2.06 |
| 96 | PSAT1 | 2.06 |
| 97 | CEP76 | 2.04 |
| 98 | PPP2R4 | 2.03 |
| 99 | COX4NB | 2.01 |
